# Vaginal Stump Ligation for Cervical Cancer

**Published:** 2017-10

**Authors:** Yanling YANG, Yang LIU, Guilin LI, Lei LI, Peng GENG, Hongjuan SONG

**Affiliations:** Dept. of Gynaecology, Xuzhou Maternal & Child Health Care Hospital, Xuzhou, China

**Keywords:** Vaginal stump ligation, Cervical cancer, Laparoscopic surgery

## Abstract

**Background:**

We aimed to investigate the effect of vaginal stump ligation in laparoscopic cervical cancer surgery on the prevention of cancer cell detachment.

**Methods:**

The study was conducted from 2010 to 2015, in Xuzhou Maternity and Child Health Care Hospital, Jiangsu Province, China. Seventeen cases of laparoscopic surgery of cervical cancer in control group were observed, and the vaginal stump was irrigated with normal saline after the operation and the washing fluid was searched for cancer cells. Moreover, 43 cases of cervical cancer patients received the same operational procedure, and the vaginal stump was ligated in the surgery and the vagina was incised below the ligature.

**Results:**

The number of cancer cells in the vaginal washing fluid of the experimental group was significantly more than that of the control group. Furthermore, there was no significant difference in the operation time, intraoperative blood loss, the number of pelvic lymph node dissected, vaginal resection length and parametrium resection length. By comparing the postoperative recovery indicators and complications, we found no significant difference in anal exsufflation time, the incidence of vaginal stump infection, the recovery time of postoperative urinary function and incidence of lymphocysts. Finally, there was no significant difference in the quality of life scores between the two groups.

**Conclusion:**

Vaginal stump ligation can reduce cancer cell detachment in cervical cancer surgery, and therefore can help preventing cancer cell implantation and tumor recurrence caused by cancer cell detachment.

## Introduction

Cervical cancer is the most common female reproductive system malignancy, and the main treatment for early stage cervical cancer (Ia_2_-IIa stage) is the surgery (1). Nowadays the most commonly adopted method to deal with this issue is to perform laparoscopic vaginal radical trachelectomy and pelvic lymphadenectomy or with abdominal para-aortic lymph node sampling at the same time. However, the postoperative recurrence of cervical cancer has always been the major focus and concern for the surgeons because the studies show 3% recurrence rate within a year after surgery with the negative margin. Furthermore, the late recurrence rate, 5 years after cervical cancer, is 7.8–11.1% (2). Failing of getting treatment of cervical cancer recurrence leads the patient to death within half a year to one year and only a few can survive more than 2 years (2).

There are many factors of cervical cancer recurrence and implantation of exfoliated cancer cells during the operation is one of them (3). In the open operation, when dealing with the vaginal stump, clamping the vagina with two pedicle clamps and incising the vagina below the clamp site is used to prevent the cancer cells from exfoliating into the vagina and from the tumor recurrence. However, in the laparoscopic surgery nowadays, the vagina stump cannot be clamped by pedicle clamp as in the open operation.

The purpose of this study was to investigate the effect of vaginal stump ligation in laparoscopic cervical cancer surgery on the prevention of cancer cell detachment, which leads to tumor cell recurrence caused by implantation.

## Materials and Methods

### Diagnostic criteria for cervical cancer

From June 2010 to February 2011, we performed vagina stump washing right after the laparoscopic surgery on 17 patients detected as having large number of cancer cells in the washing fluid. Furthermore, from February 2011 to September 2015, we performed the same laparoscopic operation on 43 patients diagnosed with cervical cancer in Gynecology Ward 14 and 15 in Xuzhou Maternity and Child Health Care Hospital, Jiangsu Province.

This study was approved by the ethics committee of Xuzhou Maternal & Child Health Care Hospital. Signed written informed consents were obtained from the patients and/or guardians.

All cases were diagnosed and confirmed by cervical biopsy, classified into stage Ia_2_∼IIa according to FIGO 2000 clinical staging criteria, and then operations were performed following Li Guangyi procedure (4). None of the patients had cardiac, pulmonary, liver or renal dysfunction, endocrine diseases or malignancies in other parts, none of them had radiotherapy or chemotherapy history. The clinical trial conformed to the ethical standard and samples were obtained with patient’s informed consent.

In control group, 17 cases of cervical cancer cases were taken and the laparoscopic surgery was performed. After the operation, the vaginal stump was washed with 200 mL normal saline and cancer cells in the washing fluid were searched. In the experimental group, 43 cases were taken and the same laparoscopic surgery procedure was performed on them. The vaginal stump was sutured in the surgery and the vagina was incised below the ligation line. After the operation, the vaginal stump was washed with normal saline and cancer cells in the washing fluid were searched. The clinical data of two groups of patients is presented in Table 1.

**Table 1:** Clinical data of two groups of patients

***Group***	***Number of cases***	***Age (yr)***	***Tumor Diameter (cm)***	***Clinical stage (n)***			
				I a2	I b1	I b2	II a
Control	17	44.6±6.9	3.3±0.9	6	5	4	2
Experiment	43	45.2±7.1	3.9±1.3	17	12	10	4

### Experimental group

Although two groups were performed with the same laparoscopic surgery but in the experimental group, before the vaginal incision, the vagina was ligated with one stitch at about 4 cm below the cervical end of the vagina in order to fix the ligature. The vagina was circumcised 0.5 cm below the ligature and after suturing the vaginal stump, it is washed with 200 mL normal saline, gently agitated, sucked out and centrifuged for 15 min under constant temperature (4°C, 1500 r/min). After that, 10 mL of precipitation was collected on which Thinprep cytologic test (TCT) was performed and observed under high power objective (10×40) of an optical microscope. The view was divided into 20 fields from which the number of cancer cells in one field was counted and multiplied by 20 to calculate the total number of cancer cells in the whole view.

### Postoperative supplement therapy:

Among all the patients, if combined with the vascular embolus, poor differentiation of cancer cells, lymph node metastasis, parametrium infiltration or cancer cells in the vaginal margin, chemotherapy was mainly performed after surgery with appropriate radiotherapy and immunotherapy. In the control group, 4 cases mainly received chemotherapy, 1 case mainly received radiotherapy and 12 cases did not receive supplement therapy. In the experimental group, 7 cases mainly received chemotherapy, 2 cases mainly received radiotherapy and 34 cases did not receive supplement therapy after surgery.

### Follow-up

All patients were followed up after discharge using outpatient follow-up, follow-up letters (E-mail), follow-up visits and follow-up calls etc. Recurrence and death of patients were recorded considering life period of the patient as follow-up period. The postoperative quality of life of all patients was scored using the Quality of Life Questionnaire-Core 30 (EORTC QLQ-C30) (5) of the European Organization for Research and Treatment of Cancer.

### Statistical analysis

Statistical analysis was performed using SPSS 17.0 (Chicago, IL, USA). The measurement data was expressed as mean ± standard deviation and the enumeration data was expressed as the percentage. The mean value of the measurement data was compared using the *t*-test of group design or Wilcoxon rank sum test. Furthermore, Chi-square test was used for the comparison between groups. Survival analysis using Log-rank test was also conducted. The significance level for all above test was considered as 5%.

## Results

### Recurrence of tumors

The results showed no recurrence of tumors in the 43 patients of the experimental group. However, in the control group, 2 cases in the 17 patients which did not have vaginal stump ligation, were found with the recurrence of tumor after 3 to 4 yr of operation on the right side of the vaginal stump.

### Number of cancer cells in the vaginal flushing fluid

Based on the *t*-test (Table 2), the number of cancer cells in the vaginal flushing fluid of the experimental group were significantly less than that of the control group (*P* <0.05).

**Table 2:** Cancer cell number in the washing fluid

***Group***	***n***	***Mean±S.D***	***t***	***P***
Control	43	46.51±22.56	−5.645	0.000
Experiment	17	82.35±21.07		

### Comparison of indicators during operation

There was no significant difference between the two groups in operation time, intraoperative blood loss, the number of pelvic lymph node removed, the length of vaginal resection, the length of parametrium resection (Table 3).

**Table 3:** Operation indicators of two groups

***Group***	***Operation time (min)***	***Intraoperative blood loss (mL)***	***Number of pelvic lymph node removal***	***Vaginal resection length (cm)***	***Parametrium resection length (cm)***
Control	226.3±55.6	321.5±203.8	16.2±4.7	3.6±0.6	3.5±0.6
Experiment	221.7±45.2	329.3±232.6	15.8±5.1	3.7±0.7	3.4±0.7
*t*	0.33	−0.12	0.28	−0.52	0.52
*P*	>0.05	>0.05	>0.05	>0.05	>0.05

### The comparison of postoperative recovery indicators and complications between the two groups

There was no significant difference in anal exsufflation time, vaginal stump infection rate, postoperative urinary function recovery time and incidence rate of lymphocyst between the two groups (Table 4).

**Table 4:** The comparison of postoperative recovery indicators and complications between the two groups

***Group***	***Anal exsufflation time***	***Urinary function recovery time***	***Incidence rate of vaginal stump infection %***	***Incidence rate of lymphocyst %***
Control	2.0±0.4	0.9±0.4	11.7	17.6
Experiment	1.9±0.5	0.8±0.3	14.0	18.6
t/χ^2^	0.74	1.06	0.05	0.01
*P*	>0.05	>0.05	>0.05	>0.05

### The comparison of long-term efficacy of the two groups

Results of quality of life showed that there was no significant difference in the quality of life scores between the two groups (Table 5).

**Table 5:** Comparison of quality of life

***Variable***	***Control group***	***Experimental Group***
Physical function	76.15±12.50	75.85±13.21
Role function	66.78±15.49	67.15±16.54
Emotions	67.50±14.75	66.82±18.74
Cognitive function	68.64±14.75	68.96±16.55
Social function	58.83±15.24	57.73±11.52
Fatigue	45.22±12.85	44.91±10.77
Nausea and vomiting	29.04±17.19	30.21±15.36
Pain	45.79±14.51	44.15±13.58
Shortness of breath	32.91±9.90	33.29±11.02
Insomnia	38.51±14.45	37.99±12.39
Loss of appetite	50.53±14.78	51.24±17.84
Constipation	42.95±20.28	42.01±19.71
Diarrhea	29.29±8.72	30.22±7.82
Economic difficulties	54.29±10.55	53.36±9.84
Overall score	48.46±15.68	49.02±16.02

## Discussion

The high incidence of cervical cancer has become one of the most important global public health issues (6). At present, early stage cervical cancer (clinical stage Ia_2_-IIa) patients without severe complications or surgical contraindications preferred surgical treatment (1). In the past, in open surgeries of cervical cancer in order to reduce the detachment of cancer cells into the vagina, the vagina was clamped with pedicle clamps and incised below the clamping site. However, in laparoscopic surgeries, the vaginal stump cannot be clamped as in the case of open surgery.

In fact, we are concerned about the implantation of exfoliated cancer cells into the vaginal stump. We are looking for exfoliated cancer cells in vagina washing fluids since there are no previous references about vaginal stump cancer cell numbers after laparoscopic surgery. Therefore, our study may be used as a reference about exfoliated cancer cell numbers in the vagina stump after laparoscopic surgery.

We found no significant difference in blood loss, operation time, postoperative recovery, the incidence of complications between the control and experimental groups, which showed that the vaginal stump ligation had no harmful effects on patients undergoing cervical cancer operation.

The main route of metastasis of cervical cancer is the direct spread and lymph node metastasis (7). The impact of laparoscopic surgery on the implantation and metastasis of cervical cancer cells may be complex and multifaceted, therefore, we should take into account not only the possible effects of the carbon dioxide pneumoperitoneum environment and the specific surgical procedures, but also the effects of surgery on the biological behavior of tumor cells themselves such as implantation and metastasis potential (8–10).

In our study, the difference in the number of vaginal stumps exfoliated cancer cells was statistically significant. Since the cervical cancer cells can be in direct contact with the vaginal incision and cause implantation metastasis, which is one of the possible ways of direct implantation metastasis in laparoscopic surgeries. The biological characteristics of tumor cells in which invasive and metastatic characteristics are the most important for the metastasis and recurrence of tumors; (11, 12) are growth autonomy, transplant ability, dedifferentiation, invasion, and metastasis. The first step of the implantation and metastasis of cervical cancer cells is the exfoliation of cancer cells from the primary site. The next step, performed in our study, is to look for changes in the expression of E-cadherin, MMP-2, VEGF-C and CD44v6 in cancer cells exfoliated from the vaginal stump and literature show (13–15) that these are closely related to cancer cell invasion and metastasis. Furthermore, it also confirms the significance of the vaginal stump ligation in reducing cervical cancer cells implantation and the recurrence of cervical cancer from the cytokine level.

## Conclusion

Vaginal stump ligation can reduce cancer cell detachment in cervical cancer surgery, and therefore can help preventing cancer cell implantation and tumor recurrence caused by cancer cell detachment.

## Ethical considerations

Ethical issues (Including plagiarism, informed consent, misconduct, data fabrication and/or falsification, double publication and/or submission, redundancy, etc.) have been completely observed by the authors.

## References

[1] Undurraga M Loubeyre P Dubuisson JB Schneider D Petignat P (2010). Early-stage cervical cancer: is surgery better than radiotherapy? Expert Rev Anticancer Ther, 10(3): 451–460. 20214525 10.1586/era.09.192

[2] Hong JH Tsai CS Lai CH Chang TC Wang CC Chou HH Lee SP Hsueh S (2004). Recurrent squamous cell carcinoma of cervix after definitive radiotherapy. Int J Radiat Oncol Biol Phys, 60(1): 249–257. 15337563 10.1016/j.ijrobp.2004.02.044

[3] Curet MJ (2004). Port site metastases. Am J Surg, 187(6): 705–712. 15191862 10.1016/j.amjsurg.2003.10.015

[4] Li GY (2006). Practical Gynecologic Laparoscopy Surgery (M). Bei Jing: People's Medical Publishing House, 302–338.

[5] Madhusudhan C Saluja SS Pal S Ahuja V Saran P Dash NR Sahni P Chattopadhyay TK (2009). Palliative stenting for relief of dysphagia in patients with inoperable esophageal cancer: impact on quality of life. Dis Esophagus, 22(4): 331–336. 19473211 10.1111/j.1442-2050.2008.00906.x

[6] Tao L Han L Li X Gao Q Pan L Wu L Luo Y Wang W Zheng Z Guo X (2014). Prevalence and risk factors for cervical neoplasia: a cervical cancer screening program in Beijing. BMC Public Health, 14: 1185. 25410572 10.1186/1471-2458-14-1185 PMC4256817

[7] Ramdass B Chowdhari A Koka P (2013). Cancer-initiating cells as target for prevention of recurring disease etiology: role of these malignant putative progenitor cells in relapse or metastasis of human cervical carcinoma. J Stem Cells, 8(3–4): 233–251. 24699026

[8] Agostini A Carcopino X Franchi F Cravello L Lecuru F Blanc B (2003). Port site metastasis after laparoscopy for uterine cervical carcinoma. Surg Endosc, 17(10): 1663–1665. 12915964 10.1007/s00464-002-9278-8

[9] Picone O Aucouturier JS Louboutin A Coscas Y Camus E (2003). Abdominal wall metastasis of a cervical adenocarcinoma at the laparoscopic trocar insertion site after ovarian transposition: case report and review of the literature. Gynecol Oncol, 90(2): 446–449. 12893216 10.1016/s0090-8258(03)00271-3

[10] Martinez-Palones JM Gil-Moreno A Perez-Benavente MA Garcia-Gimenez A Xercavins J (2005). Umbilical metastasis after laparoscopic retroperitoneal paraaortic lymphadenectomy for cervical cancer: a true port-site metastasis? Gynecol Oncol, 97(1): 292–295. 15790481 10.1016/j.ygyno.2004.11.056

[11] Rauvala M Aglund K Puistola U Turpeenniemi-Hujanen T Horvath G Willen R Stendahl U (2006). Matrix metalloproteinases-2 and -9 in cervical cancer: different roles in tumor progression. Int J Gynecol Cancer, 16(3): 1297–1302. 16803520 10.1111/j.1525-1438.2006.00448.x

[12] Uren A Fallen S Yuan H Usubutun A Kucukali T Schlegel R Toretsky JA (2005). Activation of the canonical Wnt pathway during genital keratinocyte transformation: a model for cervical cancer progression. Cancer Res, 65(14): 6199–6206. 16024621 10.1158/0008-5472.CAN-05-0455

[13] O-charoenrat P Sarkaria I Talbot SG (2008). SCCRO (DCUN1D1) induces extra-cellular matrix invasion by activating matrix metalloproteinase 2. Clin Cancer Res, 14(21): 6780–6789. 18980971 10.1158/1078-0432.CCR-08-0719

[14] Wood SL Pernemalm M Crosbie PA Whetton AD (2014). The role of the tumor-microenvironment in lung cancer-metastasis and its relationship to potential therapeutic targets. Cancer Treat Rev, 40(4): 558–566. 24176790 10.1016/j.ctrv.2013.10.001

[15] Siegel RL Miller KD Jemal A (2015). Cancer statistics, 2015. CA Cancer J Clin, 65(1): 5–29. 25559415 10.3322/caac.21254

